# Norfloxacin—Toxicity for Zebrafish (*Danio rerio*) Focused on Oxidative Stress Parameters

**DOI:** 10.1155/2014/560235

**Published:** 2014-03-13

**Authors:** Marta Bartoskova, Radka Dobsikova, Vlasta Stancova, Ondrej Pana, Dana Zivna, Lucie Plhalova, Jana Blahova, Petr Marsalek

**Affiliations:** Department of Veterinary Public Health and Animal Welfare, Faculty of Veterinary Hygiene and Ecology, University of Veterinary and Pharmaceutical Sciences Brno, Palackého třída 1/3, 61242 Brno, Czech Republic

## Abstract

The aim of the study was to investigate the effects of subchronic exposure of zebrafish (*Danio rerio*) to a fluoroquinolone norfloxacin, using selected oxidative stress parameters as a target. Toxicity tests were performed on zebrafish according to the OECD Guidelines number 203 and number 215. In the Subchronic Toxicity Test, a significant (*P* < 0.01) increase in the activity of glutathione peroxidase, glutathione S-transferase, and catalase was found. In the test, norfloxacin did not affect lipid peroxidation and catalytic activity of glutathione reductase. From the results, we can conclude that norfloxacin has a negative impact on specific biochemical processes connected with the production of reactive oxygen species in fish tested.

## 1. Introduction

Fluoroquinolone antibacterial agents are widely used for the treatment of various infections, especially against gram-negative bacteria [1]. Although this type of use leads to an entry of these compounds into the environment through the excretion of unmetabolised quinolones and the disposal of unused drugs, the main source of aquatic compartments pollution with these drugs is their use in aquaculture [2].

Fluoroquinolones are among the antimicrobial chemotherapeutics frequently detected in the aquatic environment in relatively high concentrations ranging from ng L^−1^ to *μ*g L^−1^. Their ubiquitous presence has been reported, for example, in waste water treatment plant influents [3], as well as groundwater [4], surface waters [5], and even in drinking water [6].

These drugs are rather resistant to microbial degradation [7] and may persist in water bodies because of their strong sorption properties. Photodegradation is expected to play an important role in fluoroquinolone fate in some sunlit surface waters [8]. Also chemical oxidation may be significant for their degradation [9].

Norfloxacin is a synthetic chemotherapeutic agent usually used to treat urinary tract infections [10, 11]. Norfloxacin belongs to the third generation of quinolones. The mechanism of norfloxacin action is the inhibition of DNA gyrase (a type II topoisomerase), which is an essential bacterial enzyme [12]. NOEC (no observed effect concentration) for norfloxacin was determined to be 10.38 *μ*g L^−1^ by long-term bioluminescence inhibition assay with* V. fischeri* [2]. The EC_50_ and NOEC of this compound for* Chlorella vulgaris* are 10.4 and 4.1 mg L^−1^, respectively [13].

The aim of this study was to investigate the subchronic effect of norfloxacin on zebrafish (*Danio rerio*). For the determination of norfloxacin effects, selected oxidative stress parameters such as glutathione peroxidase, glutathione reductase, glutathione S-transferase, catalase, and lipid peroxidation were used as a target.

## 2. Materials and Methods

In the study, two tests of norfloxacin were performed. As the model organism, zebrafish (*Danio rerio*) was used. The first test was performed according to the OECD Guideline number 203 (Fish, Acute Toxicity Test) [14] and the second one was performed according to the OECD Guideline number 215 (Fish, Juvenile Growth Test) [15].

### 2.1. Acute Toxicity Test

Acute Toxicity Test was performed according to OECD number 203. Norfloxacin of ≥98.0% chemical purity (Sigma-Aldrich, Czech Republic) was dissolved in water with the addition of solvent, dimethyl sulfoxide (DMSO). The final concentration of DMSO was 0.1% in each test aquarium. For the test, we used five ascending concentrations of norfloxacin (0.05, 1, 5, 10, and 30 mg L^−1^), all in a duplicate and two control groups (only water and water with a solvent). After 7 days of acclimatization, ten juvenile fish (aged 30 days) were placed in each 4 liter glass aquarium. A total of 120 zebrafish were used in the test. Food had been withheld 72 hours before the test; during the test fish were not fed. The test was performed using a semistatic method with the solutions renewed every 24 hours. Fish conditions and a number of dead fish were checked every 24 hours. Duration of the test was 96 hours. Water temperature, pH, and oxygen saturation were monitored every 24 hours and were as follows: temperature 23 ± 0.5°C, oxygen concentrations above 60% (ranged from 85% to 96%), and pH ranged from 8.28 to 8.61.

Water samples were collected for the measurement of the real concentration of norfloxacin in water every 48 hours. The concentrations of the test substance were measured using HPLC with photometric detection. Norfloxacin concentration did not fall under 90% of the nominal value.

The aim of the acute toxicity test was to determine the range of concentrations for a subsequent Subchronic Toxicity Test.

### 2.2. Subchronic Toxicity Test

Subchronic Toxicity Test of norfloxacin was performed according to OECD Guideline number 215. In the test, juvenile (30 days old) zebrafish (*Danio rerio)* were used. Fish had been acclimatized for 14 days before the test started.

Norfloxacin was dissolved in water using DMSO in volume of 2 *μ*L L^−1^ as a solvent. Fish were exposed to five ascendant sublethal concentrations, that is, 0.0001 (an environmental concentration), 0.1, 1, 10, and 30 mg L^−1^ (all in a duplicate). In the test, two control groups (the first one with only water, the second one with water and solvent, DMSO) were used. Fish were randomly distributed into 30 liter glass aquaria, 50 specimens in each aquarium. A total of 600 fish were used in the test.

The experiment was conducted in a flow-through system with a test solution renewal every twelve hours. During the test, water condition parameters were monitored at 24-hour intervals. Water quality values were measured as follows: temperature 23.7–24.6°C, oxygen saturation above 60% (ranged between 80% and 94%), and pH 8.19–8.60. In the course of the test, fish were fed with dried* Artemia salina* without shells at 8% of body weight per day. The food ration was based on an initial fish weight and recalculated after 14 days of the test. Duration of the test was 28 days.

Water samples were collected every 7th day to measure real concentrations of norfloxacin in water. Analyzed concentrations of norfloxacin in water were found to be above 92% of the nominal concentrations in the course of subchronic test, which is in accordance with the test validation criteria.

At the end of the test, fish were euthanased and then weighed. Their tank-average specific growth rates were determined. Tank-average specific growth rates were calculated using the following formula according to OECD method number 215:

(1) $$\begin{array}{c} r=\frac{\bar{\text{lo}{\text{g}}_{e}W_{2}}-\bar{\text{lo}{\text{g}}_{e}W_{1}}}{t_{2}-t_{1}}*100, \end{array}$$

where *r* is the tank-average-specific growth rate; *W*_1_, *W*_2_ are the weights of a particular fish at times *t*_1_ and *t*_2_, respectively; $\bar{\text{lo}{\text{g}}_{e}W_{1}}$ is the average of the logarithms of the values *W*_1_ for the fish in the tank at the start of the study period; $\bar{\text{lo}{\text{g}}_{e}W_{2}}$ is the average of the logarithms of the values *W*_2_ for the fish in the tank at the end of the study period; *t*_1_, *t*_2_ is the time (days) at the start and end of the study period.

### 2.3. Fish Sampling and Homogenization

At the end of the Subchronic Toxicity Test, fish were euthanased by approved anesthetic (MS222). Body weight and length of each fish were recorded. Fish were frozen and stored at –85°C until homogenization. Whole body samples were weighed and homogenized (1 : 10 w/v) using a phosphate buffer (pH 7.2). The homogenate was then divided into two parts. The first one was used for the measurement of thiobarbituric acid reactive substances (TBARS), the second was centrifuged (10,500 g at 4°C for 20 min), and the obtained supernatant fraction was then used for the determination of catalytic activities glutathione peroxidase (GPx), glutathione reductase (GR), glutathione S-transferase(GST), and catalase (CAT).

### 2.4. Measurement of Oxidative Stress Parameters

To determine a lipid peroxidation in fish samples, the TBARS (thiobarbituric acid reactive substances) method described by Lushchak et al. (2005) was used. TBARS were measured at 535 nm and the concentration was expressed in nmol of TBARS per gram of tissue wet weight [16].

The catalytic activity of glutathione peroxidase and glutathione reductase was determined spectrophotometrically at 340 nm by catalysis conversion of oxidized glutathione (GSSG) to reduced glutathione (GSH) for the consumption of NADPH. The specific activities were expressed as the nmol of NADPH consumption per min per mg of protein [17].

Total catalytic activity of glutathione S-transferase was determined by the measurement of the conjugation of 1-chloro-2,4-dinitrobenzene with reduced glutathione (GSH) at 340 nm [18]. The specific activity was expressed in the units of nmol of formed product per min per mg of protein.

For the assessment of GST and GR catalytic activities, the concentration of proteins was determined by Bicinchoninic Acid Protein Assay Kit (Sigma-Aldrich, St. Louis, MO), in which bovine serum albumin was used as a standard [19].

The catalytic activity of catalase was determined by a spectrophotometrical measurement of H_2_O_2_ breakdown at 240 nm. The specific activity of the enzyme was expressed as *μ*mol of decomposed H_2_O_2_ per min per mg of protein [20].

All spectrophotometric measurements were performed using the Varioskan Flash Spectral Scanning Multimode Reader (Thermo Fisher Scientific Inc., USA).

### 2.5. Determination of Norfloxacin Concentration in Water

Measurement of norfloxacin was based on high-performance liquid chromatography coupled with triple quadrupole tandem mass spectrometry (LC-ESI-MS/MS). Samples were filtered and used for LC-ESI-MS/MS analysis. A Thermo Scientific UHPLC Accela 1250 system was connected to a Thermo Scientific TSQ Quantum Access MAX Triple Quadrupole Instrument (Thermo, San Jose, CA, USA) equipped with heated electrospray ionization (HESI-II) probe. A Thermo Scientific Hypersil C_18_ (2.1 mm × 50 mm, 1.9 *μ*m) column was used at a constant flow rate of 300 *μ*L min^−1^. Mobile phase consisted of water containing 0.1% formic acid (v/v) (solvent A) and acetonitrile containing 0.1% formic acid (solvent B). The full loop injection volume of the tissue extract was set at 20 *μ*L. The heated electrospray ionization was operated in the positive-ion mode under specific conditions: standard of norfloxacin was purchased from Sigma-Aldrich (St. Louis, MO). All solvents were residual analysis purity (Chromservis, Ltd., CZ). Coefficient of variation for between-series was 3.1%. The limit of detection was 91 ng L^−1^.

### 2.6. Statistical Analysis

Oxidative stress biomarkers were tested for a normal distribution using the Shapiro-Wilk test. After testing for homogeneity of variance across groups, an analysis of variance (one-way ANOVA) was used. The differences among test groups were assessed with the Tukey-HSD test with *P* < 0.05 as the level of significance.

### 2.7. Ethical Statement

All experimental procedures were approved by the institutional committee and performed in a compliance with institutional guidelines and national legislation (Act number 246/1992 Coll., on the Protection of Animals Against Cruelty, as amended).

## 3. Results 

No significant difference was found between the results of control group with only water and the control one with water and the addition of a solvent (DMSO). Therefore, we used only one average value for the control group.

### 3.1. Mortality of Fish in Subchronic Test

Mortality of juvenile fish was found to be less than 8% in all experiment groups. In the control group, mortality did not exceed 4%, which is in an agreement with the validation criteria of the juvenile growth test.

### 3.2. Growth Rate

At the end of the Subchronic Toxicity Test, all fish were weighed and their length was measured. Statistical analysis of the somatic parameter was then performed.

**Figure 1:**
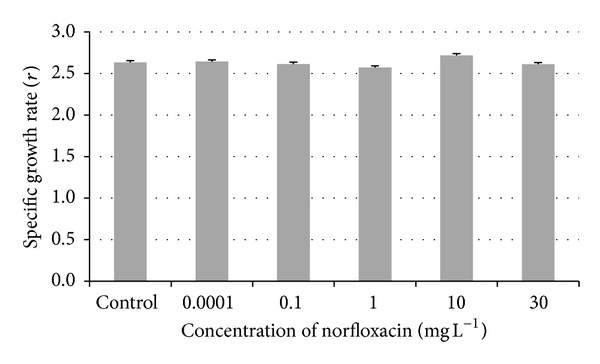
Zebrafish specific growth rate (*r*) in subchronic toxicity test (values in mean ± SEM); SEM = standard error of mean.

The specific growth rate *r* was calculated for all tested concentrations and control. No significant differences were found among the groups tested (Figure 1).

### 3.3. Effect of Norfloxacin on Biotransformation and Antioxidant Enzymes

**Figure 2:**
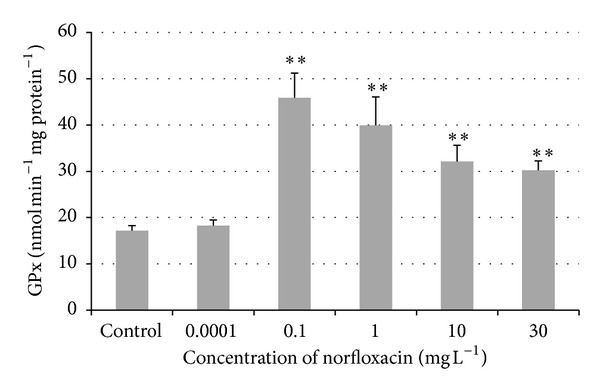
Glutathione peroxidase (GPx) activity in zebrafish exposed to norfloxacin (values in mean ± SEM); SEM = standard error of mean; ∗∗ = a significant increase in GPx activity (*P* < 0.01).

**Figure 3:**
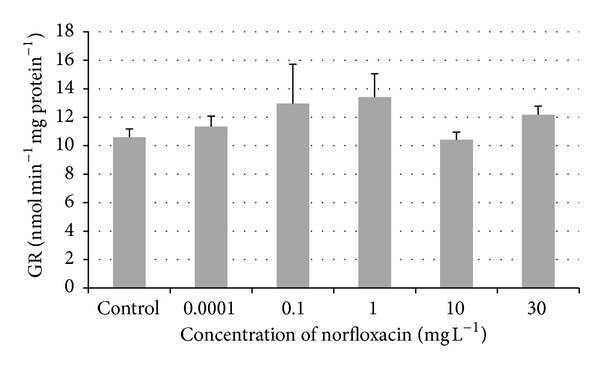
Glutathione reductase (GR) activity in zebrafish exposed to norfloxacin (values in mean ± SEM); SEM = standard error of mean.

An increase in glutathione peroxidase (GPx) activity was found in fish exposed to all concentrations of norfloxacin (0.0001, 0.1, 1, 10, and 30 mg L^−1^) compared to the control group (Figure 2). But only in the groups exposed to norfloxacin concentrations of 0.1, 1, 10, and 30 mg L^−1^ (45.86, 39.92, 32.13, and 30.24 nmol min^−1 ^ mg protein^−1^) GPx activity was significantly higher (*P* < 0.01) compared to the control group (17.16 nmol min^−1 ^ mg protein^−1^). The increase in the activity of glutathione reductase (GR) was found in norfloxacin concentrations of 0.0001, 0.1, 1, and 30 mg L^−1^ (11.33, 12.96, 13.41, and 12.18 nmol min^−1^  mg protein^−1^) compared to the control group (10.59 nmol min^−1 ^ mg protein^−1^), but the increase did not reach significance (Figure 3).

**Figure 4:**
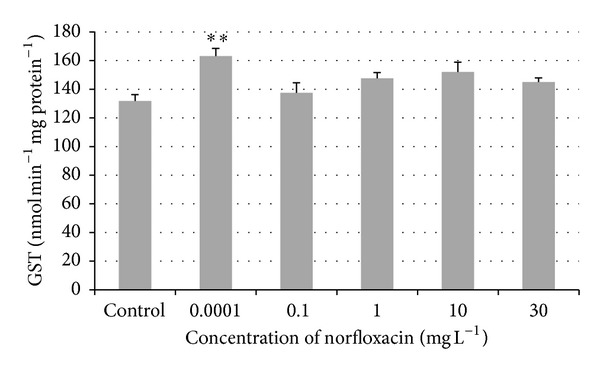
Glutathione S-transferase (GST) activity in zebrafish exposed to norfloxacin (values in mean ± SEM); SEM = standard error of mean;∗∗ = a significant increase in GST activity (*P* < 0.01).

The activity of glutathione S-transferase (GST) increased in all tested groups exposed to 0.0001, 0.1, 1, 10, and 30 mg L^−1^ (163.16, 137.49, 147.64, 152.06, and 145.03 nmol min^−1 ^ mg protein^−1^) of norfloxacin compared to the control group (131.86 nmol min^−1 ^ mg protein^−1^). The highest and significantly different (*P* < 0.01) value of GST activity was found in the environmental concentration 0.0001 mg L^−1^ compared to the control group (Figure 4).

**Figure 5:**
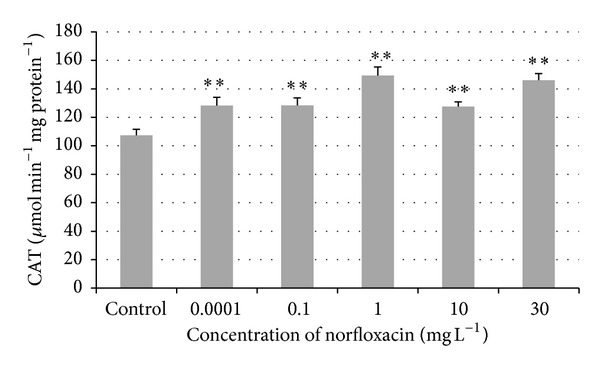
Catalase (CAT) activity in zebrafish exposed to norfloxacin (values in mean ± SEM); SEM = standard error of mean; ∗∗ = a significant increase in CAT activity (*P* < 0.01).

A significant (*P* < 0.01) increase in catalase (CAT) activity was found in all tested concentrations compared to the control group (Figure 5). In the concentrations of 0.0001, 0.1, 1, 10, and 30 mg L^−1^, catalase activity was found to be 128.29, 128.37, 149.35, 127.53, and 145.96 *μ*mol min^−1^ mg protein^−1^. In the control group, its activity was found to be 107.36 *μ*mol min^−1 ^mg protein^−1^.

### 3.4. Effect of Norfloxacin on Lipid Peroxidation (TBARS)

**Figure 6:**
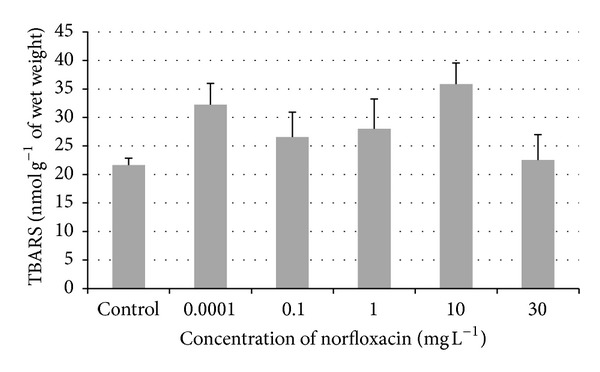
TBARS concentrations in zebrafish in Subchronic Toxicity Test (values in mean ± SEM); SEM = standard error of mean.

No significant difference was found in all tested concentrations −0.0001, 0.1, 1, 10, and 30 mg L^−1^ (32.22, 26.56, 28.01, 35.86, and 22.54 nmol g^−1^ of wet weight) of norfloxacin when compared to the control group (21.63 nmol g^−1^ of wet weight). In our study, norfloxacin did not affect the lipid peroxidation (Figure 6).

## 4. Discussion

In the literature, there is little information on the effect of norfloxacin (or other fluoroquinolones) on fish and other water organisms; therefore, we had to compare our results with the effect of other pharmaceuticals on fish.

In our study, we did not find any significant differences in growth rates in zebrafish exposed to all norfloxacin concentrations when compared to the control group. These results disagree with the findings of Nie et al.'s study [21], in which the effect of norfloxacin on the freshwater microalga (*Scenedesmus obliquus*) was evaluated. Nie et al. proved that the concentrations used in the test (0.0–60.0 mg L^−1^) inhibited growth rates of tested alga, which can be probably explained as a direct toxic effect of norfloxacin, an antimicrobial agent, to unicellular alga. The results of his study demonstrated that the concentration resulting in 50% inhibition of algal growth rate after 48 hours of exposition (48 h IC_50_) was found to be 38.49 mg L^−1^ [21]. In the study on the subchronic effect of diclofenac (a nonsteroidal anti-inflammatory drug, NSAID) on early stages of common carp (*Cyprinus carpio*), no effect on body weight and growth was found in experimental fish compared to control [22]. This result is in an agreement with our results. In the study of Zivna et al. [23], toxic effect of acetylsalicylic acid (NSAID) in the concentration range from 0.004 to 250 mg L^−1^ on zebrafish was tested and the increase in body weight and specific growth rate was found in all experimental groups compared to the control group.

Glutathione peroxidase (GPx) is an enzyme which transforms hydroperoxides to hydroxyl compounds using a reduced glutathione as a substrate. In our study, a significant increase (*P* < 0.01) was found in the activity of glutathione peroxidase in zebrafish exposed to norfloxacin at the concentrations of 0.1, 1, 10, and 30 mg L^−1^. It can be explained by the presence of oxidative substances in cells, which may cause an increase in antioxidant enzymes activities as a defense mechanism [24]. Environmental concentration of norfloxacin (0.0001 mg L^−1^) did not cause an increase in the activity of GPx in our experiment. The increase in GPx activity was also found in the study performed on zebrafish exposed to ibuprofen (NSAIDs) in the concentrations of 0.05, 1, 8, and 25 mg L^−1^ [25].

Glutathione reductase (GR) is an enzyme catalyzing the conversion of glutathione disulfide to reduced glutathione. The main function of reduced glutathione is the protection of cells from chemical insult [26, 27]. In our study, we did not find significant differences in glutathione reductase activity in all concentrations tested. On the contrary, in the study of Zivna et al. [23], the exposure of zebrafish to acetylsalicylic acid in the concentrations of 0.004, 0.4, 40, 120, and 250 mg L^−1^ caused a significant increase in the activity of GR when compared to the control group.

The main function of glutathione S-transferase (GST) in the endogenous metabolism is the detoxification of xenobiotics and products of oxidative stress. GST provides cellular protection against toxic effects of a variety of environmental and endogenous chemicals [27]. The exposure of zebrafish to norfloxacin caused a nonsignificant increase in GST activity in all tested groups compared to the control group. A significant (*P* < 0.01) increase was found only in the fish exposed to environmental concentration (0.0001 mg L^−1^). In freshwater microalga (*Scenedesmus obliquus*) exposed to norfloxacin in the concentration range between 0.0 and 60 mg L^−1^, GST activity was found to be significantly increased at higher norfloxacin concentrations, reaching a peak value at 15 mg L^−1^ [21]. Oliveira et al. [28] investigated the effect of two antibiotics (oxytetracycline and amoxicillin) on zebrafish enzymes. GST activity was induced in muscle and gill samples. On the contrary, in head samples an inhibition trend was observed. In zebrafish exposed to oxytetracycline, GST activities were increased at almost all concentrations above 1 mg L^−1^ in muscle and liver samples. GST activity in zebrafish exposed to 40, 120, and 250 mg L^−1^ of acetylsalicylic acid in the study of Zivna et al. [23] was also found to be significantly higher. Stepanova et al. [22] studied the effects of diclofenac on early stages of common carp and found a significant increase in the GST activity at the highest tested concentration of diclofenac (3 mg L^−1^).

Catalase is an enzyme that catalyzes the conversion of a potentially oxidative molecule, H_2_O_2_ into H_2_O and O_2_ [29]. In our study, significantly higher catalase activity was found in all tested concentrations (0.0001, 0.1, 1, 10, and 30 mg L^−1^) compared to control. In the study of Wang et al., the toxicity of fluoroquinolone enrofloxacin associated with environmental stress in Tra catfish (*Pangasianodon hypophthalmus*) was tested. Fish were fed with pellets containing 1 g kg^−1^ enrofloxacin for 7 days. A 1.7 times increase in CAT activity in gills of the fish exposed to enrofloxacin was found in a low-density group (40 fish m^−3^) contrary to one and three days after the end of exposure (day 8 and 10); CAT activity was significantly lower than in the control group [29]. In the study of Oliveira et al., amoxicillin at the highest concentration (221 mg L^−1^) inhibited the activity of CAT in gills and head samples of zebrafish. Zebrafish exposed to oxytetracycline in the concentration of 25.0–100.0 mg L^−1^ exhibited a dose-dependent inhibition of CAT activity in head samples, whereas no alteration was observed in the other tissues analyzed [28].

The level of TBARS is used to measure the extent of lipid peroxidation. One of the major terminal products of lipid peroxidation is malondialdehyde [30, 31]. Lipid peroxidation has been a major contributor to the loss of cell function under oxidative stress [32]. In our study, we did not find any significant effect of norfloxacin on lipid peroxidation. In the study of Wang et al., in which the effect of enrofloxacin on Tra catfish (*Pangasianodon hypophthalmus*) in the relation to density stress was tested, lipid peroxidation in gills of enrofloxacin-fish reared at low (40 fish m^−3^) or high (120 fish m^−3^) density was significantly (more than 5-fold) higher than their respective control at day 7. On the contrary, lipid peroxidation in gills of enrofloxacin-fish reared at medium density (80 fish m^−3^) was significantly, 3-fold lower than in the control fish [29]. Juvenile rainbow trout (*Oncorhynchus mykiss*) exposed to six ascending concentrations (5, 10, 15, 20, 25, and 30 mg L^−1^) of carbamazepine (anticonvulsant drug) for 96 hrs showed an increase in lipid peroxidation (*P* < 0.05) in gill and brain when compared to the control group [33].

## 5. Conclusions

The results of our study indicate that norfloxacin does not affect the growth rate in zebrafish. Norfloxacin in the tested concentrations affects some biomarkers of oxidative stress (GPx, GST, and CAT). The subchronic exposure of zebrafish to norfloxacin causes the increase in the activities of some antioxidant and biotransformation enzymes. Based on these results, we can conclude that norfloxacin (even in environmental concentrations) may have a negative impact on some biochemical processes connected with the production of ROS (reactive oxygen species) in aquatic organisms.
